# Rapunzel Syndrome in Congenital Mental Retardation Patient Requiring Emergency Laparotomy. Case report

**DOI:** 10.1016/j.amsu.2020.08.026

**Published:** 2020-09-01

**Authors:** Abdelaziz Alibraheem, Aghyad Danial, Amira Kazan, Esraa Masri, Mohammad Yusr Obari, Samer Rajab Basha

**Affiliations:** a MD, Department of Surgery, Faculty of Medicine, University of Aleppo, Syria; b Faculty of Medicine, University of Aleppo, Aleppo, Syria

**Keywords:** Abdominal mass, Rapunzel syndrome, Trichobezoar, Trichotillomania

## Abstract

**Introduction:**

When hair accumulates inside the stomach, it causes what is called a Trichobezoar, which leads to a stomach blockage. When the accumulated hair extends into the small intestine, it causes a rare disturbance called Rapunzel syndrome (RS).

**Discussion:**

Affected patients infrequently remain asymptomatic for several years. Symptoms begin while the bezoar increases in size to the point of obstruction, these symptoms are nonspecific like vomiting, nausea, anorexia, asymptomatic abdominal mass and digestive bleeding.

**Presentation of case:**

The authors report an unusual case of a 25 years old young woman who presented with acute abdominal pain and gastrointestinal symptoms. With an upper gastroscopy, the condition was first diagnosed as a Trichobezoar that occupied the stomach. A decision to perform a surgical procedure was taken, only to discover, during the procedure, that the mass was extended to the duodenum and jejunum, thus diagnosing the condition as Rapunzel syndrome. The patient was managed by surgical removal of the huge mass.

**Conclusion:**

Trichobezoar considers as a differential diagnosis for any patient with mental retardation and has gastrointestinal symptoms.

## Highlights

• The neuropsychiatric disorder may associate with Trichophagia that is Leeds to Rapunzel syndrome.
• Surgical treatment was the golden choice of extraction of the Trichobezoar.
• The patient must not eat hair again after recovery to prevent bezoar recurrence.
• Keep the hair always too short for pulling is the only solution for the patient with congenital mental retardation.

## 1 Introduction

Our paper has been reported according to SCARE criteria [10].

Bezoar is an abnormal condition, in which non-digestible substances accumulate inside the gastrointestinal tract causing its blockage.

In the absence of adequate treatment, the associated mortality rate is up to 30%, principally because of gastrointestinal bleeding, destruction, or perforation [1].

There are various types of bezoars, one of which is Trichobezoar, in which there is a gathering of hair inside the gastrointestinal tract. This condition is more common in animals than in humans, and this type of bezoar is the most common in humans [2], Trichobezoars are associated with trichophagia as a result of pica – an eating disorder manifested by an appetite for nonnutritive substances and often associated with mental alteration – and coexistent psychiatric disturbances [3].

The word “Trichobezoar” is a combination of “trich” meaning hair in Greek and “bezoar” meaning poison antidote in Arabic or Persian [4].

Rapunzel syndrome is a rare condition of Trichobezoar when swallowed hair extends to the small intestine in addition to the stomach, it is more common in women, especially adolescents’ girl 90% [5]. It also happens during the second decade of life 4, causing a wide range of symptoms. It was named after Rapunzel, the heroine of a German fairy tale [6].

## 2 Case report

A 25- year female was referred to our surgical clinic with acute worsening abdominal pain; the pain firstly started 3 months ago as a poorly localized abdominal pain associated with postprandial emesis, nausea, anorexia, episodes of watery small-volume diarrhea, and about 6 kg of weight loss within the last month.

The physical examination revealed a malnourished girl; The patient had stable vital signs.

Abdominal examination showed a firm, non-tender, non-compressible and non-mobile mass in the epigastrium area, with irradiation to the left hypochondrium, another systemic examination was unremarkable and the laboratory investigation of the case included the following results: white blood cells: 8100/μl hemoglobin: 11.9 mg/dl hematocrit: 41.4% folic acid: 9 ng/ml ferritin: 13 ng/ml iron: 67 μg/dl iron binding capacity: 268 μg/dl with normal liver chemistries and lipase.

There were hairless regions on her scalp in the frontal and parietal areas, bilaterally.

Her parents mentioned that she had a history of eating hair and nails as a result of congenital mental retardation.

The computed tomography (CT) scan revealed a huge gastric distention with well-circumscribed and heterogeneous mass occupying the whole stomach cavity with extension into the proximal jejunum.

Upper gastrointestinal endoscopy disclosed a large hair mass occupying the whole stomach, and a diagnosis of “Trichobezoar” was made.

**Fig. 1:**
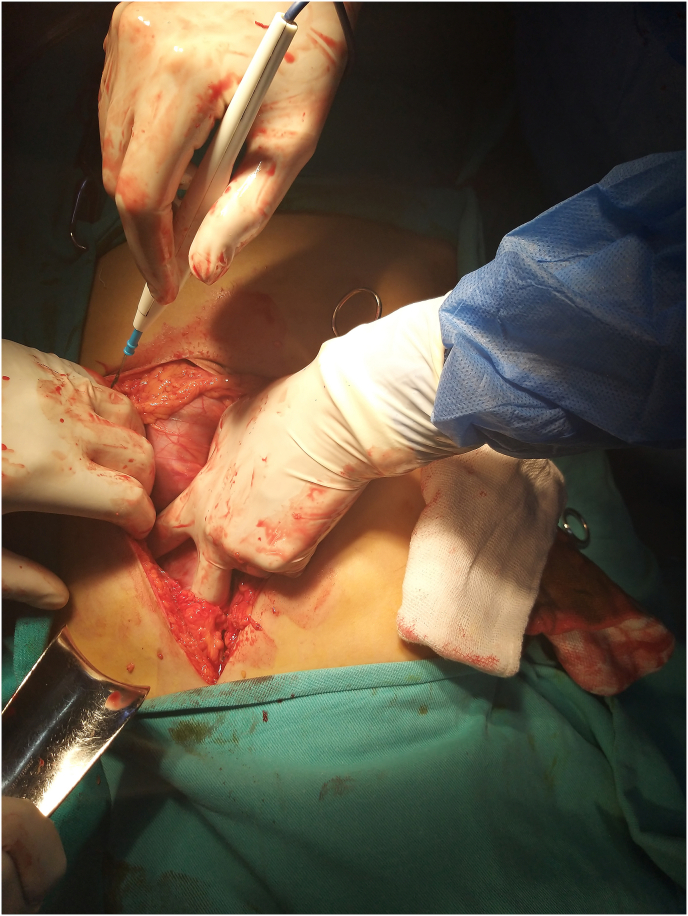
Interoperation photo.

**Fig. 2:**
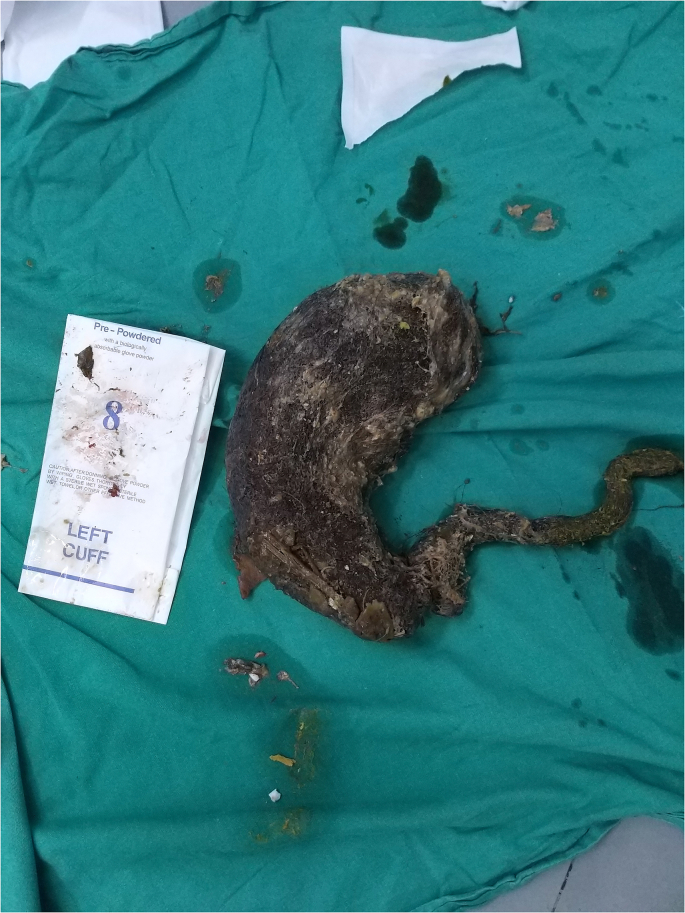
Giant trichobezoar with a long tail of hair extending within the pylorus into the proximal jejunum (Rapunzel Syndrome).

Therefore, the patient underwent emergency laparotomy; an anterior gastrotomy was done (Fig. 1). There was a giant trichobezoar with a long tail of hair extending within the pylorus into the proximal jejunum; the mass takes the shape of the stomach and duodenum. Due to this feature, “Rapunzel Syndrome” was the clear diagnosis (Fig. 2).

We closed the incision in two layers using 2-0 Vicryl suture and the abdomen was closed without drainage.

After the operation, there was no leakage. She started feeding 24 hours after the operation and was discharged after 5 days. Psychiatric treatment in this situation is pointless because our patient has congenital mental retardation and the only solution here is to keep her hair consistently too short for pulling.

## 3 Discussion

Bezoars are concretions of foreign substances in the gastrointestinal tract, mainly the stomach. Bezoars composed of hair or hair-like fibers are called 'trichobezoars'. Trichobezoars tend to appear in the second decade of life [3]. Often in females with psychiatric disorders including trichotillomania (pulling out their hair) and trichophagia (eating hair) [7].

Trichobezoars form when Ingested hair strands are accumulated in the gastric folds, escaping peristaltic propulsion because of their slippery surface, prevents enough friction which is required to push them out of the stomach. The Ingested hair becomes even more matted together and takes the shape of the stomach, usually as a single solid mass [8].

Several criteria have been used to describe “Rapunzel syndrome” some authors define it as a trichobezoar with a tail that extends through the pylorus while others indicate to it as a concomitant intestinal obstruction.

Affected patients infrequently remain asymptomatic for several years. Symptoms begin while the bezoar increases in size to the point of obstruction. The patient with a gastric trichobezoar usually presents with nonspecific symptoms, including abdominal pain (70%), nausea and vomiting (64%), digestive bleeding (61%), epigastric discomfort, early satiety, indigestion, weight loss (38%), diarrhea or constipation (32%) [3].

Complications by a huge eroding or obstructing bezoar additionally involve obstructive jaundice, severe anemia either due to malabsorption or gastrointestinal bleeding, acute pancreatitis, and gastric emphysema and this complication are infrequent and raise mortality rate to 30%.

Rapunzel syndrome diagnosis is made by endoscopic examination and radiography imaging. Upper gastrointestinal (GI) endoscopy can provide information about the structure of the mass. The Computed tomography (CT) investigation can Reveal the existence, localization, and distribution of the bezoars [9].

Different therapeutic modalities have been suggested to treat trichobezoar like endoscopy, Surgery, and pharmacological approaches. Surgery by gastrotomy or enterotomy is still the mainstay for gastric trichobezoar removal especially those that extend into the intestine. Because of the enormous size of the mass, laparotomy was chosen as the surgical method in order to remove the whole trichobezoar mass successfully.

## 4 Conclusion

Trichobezoar considers as a differential diagnosis for any patient with mental retardation and has gastrointestinal symptoms.

Small trichobezoars may be extracted by endoscopic fragmentation. Bezoars like Rapunzel syndrome, on the other hand, need surgical removal.

Early diagnosis and an appropriate therapy can reduce morbidity and mortality.

Psychological counselling plays a pivotal role in order to prevent bezoar recurrence, but our patient has Congenital mental retardation and the only solution here is to keep her hair always too short for pulling.

## Funding

This research did not receive any specific grant from funding agencies in the public, commercial, or not-for-profit sectors.

## Authors’ contributions

AD diagnosed and treated the patient,

AA and EM searched the literature,

EM, AK, SRB and MYO wrote the manuscript.

AA critically revised the article.

All authors approved the final version of the manuscript.

## 5 Ethics and consent to participate

We have the patient's approval; no more approvals are required.

The work has not been published previously.

## 6 Consent to publish

Written informed consent was obtained from the patient for publication of this case report and accompanying images. A copy of the written consent is available for review by the Editor-in-Chief of this journal on request.

## 7 Availability of data and materials

All data and materials are available.

Provenance and peer review.

Not commissioned, externally peer reviewed.

## conflicts of interest

Authors declare that there is no conflict of interest.

## Declaration of competing interest

Authors declare that there is no conflict of interest.
